# Biomarkers in T cell therapy clinical trials

**DOI:** 10.1186/1479-5876-9-138

**Published:** 2011-08-19

**Authors:** Michael Kalos

**Affiliations:** 1 Department of Pathology and Laboratory Medicines, University of Pennsylvania Perelman School of Medicine, Abramson Family Cancer Research Institute, 422 Curie Boulevard, Stellar-Chance Laboratories, Philadelphia, PA 19104-4283, USA

## Abstract

T cell therapy represents an emerging and promising modality for the treatment of both infectious disease and cancer. Data from recent clinical trials have highlighted the potential for this therapeutic modality to effect potent anti-tumor activity. Biomarkers, operationally defined as biological parameters measured from patients that provide information about treatment impact, play a central role in the development of novel therapeutic agents. In the absence of information about primary clinical endpoints, biomarkers can provide critical insights that allow investigators to guide the clinical development of the candidate product. In the context of cell therapy trials, the definition of biomarkers can be extended to include a description of parameters of the cell product that are important for product bioactivity.

This review will focus on biomarker studies as they relate to T cell therapy trials, and more specifically: i. An overview and description of categories and classes of biomarkers that are specifically relevant to T cell therapy trials, and ii. Insights into future directions and challenges for the appropriate development of biomarkers to evaluate both product bioactivity and treatment efficacy of T cell therapy trials.

## Review

### The central role for Biomarkers in clinical research

The ultimate objective for clinical trials is to evaluate the safety and efficacy of novel therapeutic agents. Although the ability to evaluate safety is in general rather straightforward, the ability to measure clinical efficacy is often compromised. The reasons for this are multiple and include the variable and often long times to progression, the fact that direct measurements on target tumors are often not possible, and also include patient- intrinsic effects related to both patient and tumor heterogeneity. Nonetheless, early evidence for product efficacy and bioactivity is of critical importance in the clinical trial process to guide the further development of the candidate product. Well-designed biomarker studies provide a primary mechanism to evaluate product efficacy and bioactivity, and also provide fundamental insights into mechanistic aspects of the treatment regimen.

The clinical development path for novel therapeutics has historically followed a rather rigid and iterative approach that has imposed certain significant limitations on the effective development of promising therapeutics, since the inherent rigidity of the approach does not allow for the flexibility to either accelerate trials when early results are particularly promising, or to modify the trial design as information and knowledge about the treatment impact, response and biomarker profile is generated (see for example [1]).

Two conceptually related proposals for clinical trial design, the adaptive [2,3] and two-stage [4] clinical trial design paradigms, have been recently proposed to overcome at least some of the limitations associated with the traditional clinical development path for new therapeutics. Both the adaptive and two-stage clinical design paradigms are integrally dependent on the development and application of robust, relevant and statistically-based biomarker studies to guide the clinical development process; accordingly, increased implementation of these approaches has fostered a renewed emphasis on the development of high quality biomarker research [5-9].

Recent focus on the establishment and implementation of integrated translational research programs has highlighted a critical role for biomarkers during preclinical stages of research. In addition to guiding go-no-go decisions to move new agents into the clinic, preclinical biomarker studies commonly evaluate mechanistic aspects of the product, and often serve to define both the biomarkers to be studied and the assays to be employed in the clinical trial. A strong argument can thus be made for the close integration of biomarker development from the preclinical through the clinical trial process.

### T cell therapy clinical trials

The concept of enhancing cellular immunity through the transfer of ex-vivo expanded T cells was pioneered by Greenberg et al., who coined the term adoptive T cell transfer to describe the process [10]. The first clinical application of adoptive T cell transfer involved reconstitution of cellular anti-CMV immunity in the context of allogeneic bone marrow transplantation [11]; since then, adoptive T cell transfer has been evaluated as a treatment modality against a number of viral diseases [12-14].

Significant effort has been put forth over the past few years to evaluate the potential to treat cancer via the adoptive transfer of T lymphocytes, both effector lymphocytes (CD8 and CD4) and regulatory (Treg) cells, manipulated ex-vivo to generate large numbers and in some cases to enhance their activity (see for examples [15-17]). Such efforts been enabled by enhanced understanding of T cell immunobiology, and facilitated by the development of approaches to expand and manipulate T cells ex vivo [18-20], methodologies to enable manufacture under Good Manufacturing Practice (GMP) [21-23], as well as genetic approaches to augment T cell specificity and function [24,25]. These developments have facilitated a broad range of clinical trials to evaluate the ability of T cell therapy-based strategies to target tumors. T cells, derived from the periphery [17,26-28], from tumor infiltrating lymphocytes (TIL) [29-31], or have been enriched for virus-specificities [13,32,33] to enhance persistence have been infused into patients after ex-vivo expansion either as bulk or antigen-specific populations. More recently, advances in the practical ability to genetically engineer T cells through retro- and lenti-virus mediated transfer of DNA into primary human T cells have opened up the opportunity to augment and re-direct anti-tumor activity through gene transfer of tumor-antigen- specific T cell receptors (TcR) [15,34,35] or chimeric antigen receptors (CAR) to manifest novel anti-tumor specificities [36-38]. Even more recently, high efficiency RNA transfer technologies have been developed to genetically engineer T lymphocytes in a transient manner [20,39]. Such "biodegradable" re-directed T cells afford the potential to effectively target tumors while minimizing the potential negative consequences associated with long-term persistence of gene-modified cells. On the other hand, due to the transient nature of the functional product, biomarker studies for RNA-modified T cells are likely to be restricted to the assessment of infusion-proximal and acute events.

To date, essentially all T cell therapy trials have been early stage trials with the primary objectives related to feasibility and safety. Although dramatic results have been observed in a number of cases, by virtue of cohort sizes such trials have only offered tantalizing hints into potential efficacy [15,40,41].

### Biomarkers in T cell therapy trials

The vast majority of to-date clinically evaluated anti-cancer products are in essence chemical compounds. This holds true for bio-molecules such as antibodies, peptide or proteins, adjuvants, small molecule agonists and antagonists, as well as radio- and chemo-therapeutic agents. Each of these product classes targets a physiological process in the tumor and/or in the patient and has a well defined half-life, but from a biological perspective is essentially inert. Accordingly, biomarker studies for such agents have focused on the impact of the treatment on the target tissue(s). Examples of such efficacy biomarkers include secreted and shed tumor products such as PSA, PSMA, her-2-neu and many others (reviewed in [42]), circulating tumor cells [43], the detection of minimal residual disease using tumor specific genetic rearrangements such as Bcr-Abl [44], and more recently tumor-specific epigenetic modifications [45].

Cell therapy trials in general and T cell trials specifically are distinguished by the fact that the product is a biological entity whose physiological status is critical to mediate the desired therapeutic effect; essentially, the transferred T cells need to be both present and functional for treatment to be efficacious. Consequently, T cell therapy trials require the development and evaluation of additional classes of biomarkers that describe the biological properties of the cell product. Accordingly, a fundamental understanding of the biomarkers that are relevant for T cell functional competence has important consequences for the ability to effectively evaluate T cell bioactivity in patients.

### Product Biomarkers for T cell trials

Results from both animal studies and clinical trials have identified biological parameters that are likely to be important for T cell bioactivity. These parameters can broadly be described in terms of i. presence, ii. relevant phenotypes and functional competence, iii. systemic impact on patient biology, and iv. patient immune responses to the infused product. A summary of the classes of T cell biomarkers together with types of established assays for each class as well as advantages and disadvantages for each assay is presented in Table 1.

**Table 1:** Categories and attributes of T cell biomarkers

Category	Platforms	Assay	Advantages	Disadvantages
Presence	Flow cytometry	Surface marker detection	Individual cells detected	Sample intensive Low sensitivity Specific detection reagent
	PCR	Transgene-specific amplification	High sensitivity	Bulk analysis
	Deep sequencing	Detection of specific TcR clonotypes	Extremely high sensitivity	Technology intensive
				
Phenotype/Function	Flow cytometry	Surface and intracellular marker detection	Individual cells detected Many markers available	Sample intensive Relevant functional markers unclear
				
Bioactivity	Flow Cytometry	Surface and intracellular marker detection	Individual cells detected	Low sensitivity Sample intensive
	Biochemical	Soluble factor detection	Multi-plexable Mechanistic	Bulk analysis Potentially indirect
	High-throughput Arrays	Transcriptional profiling Proteomic profiling Cytokine profiling	Relatively unbiased High throughput Mechanistic	High end Cost intensive
				
Immune response	Flow cytometry	Cellular and humoral immune responses	Individual responses can be characterized	Low sensitivity Often requires in vitro expansion
	ELISA	Humoral immune responses	High sensitivity	

#### i. Biomarkers to evaluate T cell presence

The presence of infused T cells in patients is most commonly described in terms of peripheral T cell persistence and homing to target tissues. For most T cell therapy trials the total amount of T cell product infused into patients is a fraction of the total patient T cell load, typically no more than 0.1% of the total. However, since most current clinical protocols that involve adoptive T cell transfer are preceded by a lympho-depleting regimen, infused T cells have the potential to be found as a significant percentage of total leukocyte counts, particularly at early time-points post transfer. In addition, because there is potential for in vivo expansion of the infused T cells due to homeostatic and/or antigen-driven expansion, it is possible that infused cells can be found in the reconstituted T cell compartment at numbers substantially higher than those infused [35,40].

The vast majority to T cell therapy trials have evaluated product biomarkers in peripheral blood, which is typically straightforward to obtain as part of routine blood sampling during the course of treatment. A compelling argument can be made, supported by recent clinical data, that it also critical to evaluate the quantity and functional quality of infused T cell products at the site of disease [46].

Presence (persistence, homing) of infused T cell products has been evaluated primarily by flow cytometry and molecular -based approaches.

Flow-cytometry-based approaches: The antigenic specificity of T cells is mediated through the α/β heterodimer which is part of the TcR complex. Accordingly, detection of specific TcR α/β pairs present on infused cells is one approach to evaluate and quantify infused T cell products. In most cases, this approach requires that the frequency of specific product cells is at least 0.2-0.5% of the total CD3+ T cell population to accommodate technical limitations of the flow-cytometry platform. For products that are composed of CD8 T cells with a defined antigenic specificity, MHC (major histocompatibility complex) class I multimers (tetramers, pentamers, dextramers) have been employed to detect and quantify infused cells. Because class II reagents have proven to be problematic to manufacture, multimer-based detection approaches have been more difficult to implement for CD4+ T cells, although recent reports suggest progress in this area [47]. This approach has been applied in a number of T cell therapy trials to both detect and quantify and infused antigen-specific T cells. As described below, this approach can be combined with more detailed phenotypic and/or functional studies to obtain more integrated data sets about the T cell product. One caveat of this methodology is that activation-induced down-modulation of the TcR complex may result in a reduced ability to detect recently activated cells.

A number of clinical trials are underway and/or planned that involve the transfer of T cells gene modified to target tumors through CAR [48]; since CAR typically contain an antibody--derived ScFv (single-chain variable fragment) component, anti-ScFv or idiotype-specific antibody reagents that recognize the CAR could be used as reagents to detect and enumerate antigen-specific T cells; a successful application of this concept to detect, quantify and study the phenotype of persisting CAR-modified T cells by multi-parameteric flow cytometry has been recently reported [40].

Another flow-cytometry-based approach to identify and track T cell products takes advantage of the wide availability of antibodies that recognize the variable segment of the TcRβ chain (Vβ). A total of 65 Vβ segments in the TcRβ locus have been identified that can be grouped into 25 Vβ families with each family representing roughly 0.2-5% of the total T cell population [49]. This approach is dependent on a monoclonal or at most oligoclonal T cell product, and a relatively high level persistence of infused cells (> 5% of total CD3+ cells) because of the normal distribution of T cells from each Vβ family in the non-modified T cell repertoire. Since the Vβ antibody reagents detect both endogenous and infused T cells with equal efficiency, definitive quantification of infused cells using this approach is not possible. This approach has been used in a number of clinical trials to evaluate T cell persistence (see for example [35,50,51]. As above, this approach is susceptible to the consequences of activation-induced receptor down-modulation.

Finally, Wang et al have recently described the development of a truncated EGFR polypeptide devoid of all known ligand-binding and signaling domains that can be co-introduced into human T cells and serve both a selection marker as well as a cell -surface tracking marker for adoptively transferred cells [52]. While such promising approaches offer the potential to bypass limitations associated with down-modulation, they do open up the possibility for immune rejection responses that target unique peptide epitopes from the modified polypeptides.

A different approach to evaluate T cell persistence has involved the use of quantitative PCR (Q-PCR). This approach is possible if the T cell product has been genetically engineered to contain transgenes, such as TcR, CAR, or selectable markers such as neomycin phosphotransferase and HyTK; in principle, if sufficient sequence information is available, this approach can also be utilized with primer/probe pairs specific for the Vβ sequence of the infused products [53]. This methodology has been applied in a number of clinical studies [36,40,41,51,54,55], and is considerably more sensitive than flow cytometry-based approaches, with an ability to detect modified cells at frequencies as low as 0.01% of total T cells. Significant limitations of this approach include the facts that data are generated from a bulk population of cells, that this approach is not readily amenable to dissecting in more detail the phenotype and function of the persisting T cell population, as well as the fact that this approach does not provide information about the expression status and function of the evaluated transgene. Notably, for biodegradable RNA-based T cell products Q-RT-PCR rather than Q-PCR must be utilized to track and quantify infused cells.

Novel technologies that enable high-throughput and deep sequencing of TcR variable and CDR3 domains from bulk PBMC [56,57] afford the opportunity to comprehensively evaluate the T cell diversity of infusion products and track directly ex-vivo the expansion, persistence and homing of infused cells with very high sensitivity.

#### ii. Biomarkers to measure biologically relevant phenotypes and functions of T cells

Over the past few years technical advancements in polychromatic flow-cytometry have enabled a substantially more detailed phenotypic and functional evaluation of T cell products. Flow cytometry analyses that simultaneously evaluate 12-marker are routinely performed in research laboratories while analyses that involve up to 17 markers can be performed by specialized laboratories [58-60]. Such analyses are dependent on the ability to identify the infused T cell product using multimers, anti-Vβ, or anti-T cell surface receptor antibodies as described above, and typically employ combinations of antibodies specific for surface markers that interrogate T cell differentiation, activation, and functional status and intracellular markers that reveal T cell functional activity. New technologies such as inductively-coupled mass spectrometry (ICP-MS) that can detect and quantify heavy-metals conjugated to individual antibodies offer the potential to simultaneously query for co-expression of large numbers of markers unencumbered by limitations associated with spectral overlap and differential emission of fluorescent molecules [61,62].

Recent data from both animal models and clinical trials have provided important insights about T cell phenotypes that may causally correlate with treatment efficacy: Data generated principally from the surgery branch at the NCI using adoptive transfer of TIL have suggested that treatment efficacy is related to the persistence of T cells that are or can convert *in-vivo *to memory cells [54,63]; such cells are capable of long term persistence, a property that may well be required for ultimate efficacy of T cell therapy. These results have been more systematically evaluated and confirmed in primate models [64], and a number of clinical trials are being planned at multiple institutions that involve the specific transfer of memory cell populations into patients.

A large variety of surface markers have been described in the literature as potential biomarkers for T cell differentiation status related to functional competence. Common markers for such analyses include T cell differentiation markers such CD45 RA or RO, CD62L, CCR7, CD27, CD28, combined with T cell activation markers such as CD25, CD127, CD57, and CD137 [65,66]. Although there is some uncertainty about what surface markers best define T cell differentiation state, commonly accepted phenotypic markers for the different subsets include the following (differentiation status phenotypes in [brackets]: CD45RO/CCR7/CD27/CD57: [naïve: -/+/+/-]; [effector memory: +/-/-/-]; [effector: -/-/+/+ and -/-/-/+]; [central memory +/+/+/-, +/-/+/-, +/-/+/+] [66].

Data from clinical trials that have evaluated the ability of vaccines to elicit a protective immune response in the infectious disease field have revealed that protective responses are also associated with the quality of the T cell response and the presence of T cells that simultaneously express multiple effector functions, defined as polyfunctional T cells [67-69]. Functional markers often evaluated include IL-2, TNF-α, IFN-γ, MIP1b and the de-granulation marker CD107, and protective responses are associated with polyfunctional T cells (both CD4 and CD8) which express high levels for each of the above factors. In addition, it is relevant to evaluate surface molecules such as CD25/CD127 associated with a suppressor T cell phenotype in CD4+ T cells (CD25++/CD127-) [70], as well as PD-1, BTLA, and TIM-3 which are associated with a state of T cell inhibition. More recent studies have revealed that cytotoxic T cells which express high levels of perforin, granzyme-B and the transcription factor T-bet are associated with protective responses in viral diseases, supporting the position that one or more of these functional markers be included in biomarker panels [71-73]. Efforts are ongoing to optimize and validate strategies that seek to evaluate memory phenotype and polyfunctionality [74]. However, embracing the to-date defined markers as defining the signature of a biologically relevant polyfunctional cell must be done with significant caution since it is extremely unlikely that the full extent of the optimal biological phenotype has been defined [75].

Studies from the NCI have revealed that telomere length was the one biomarker that consistently correlated with persistence of infused T cells [51], reflecting at least in part the concept that "younger" less differentiated cells may be more efficacious in vivo. More recently, Turtle et al. have demonstrated a surface marker phenotype for a distinct subset of T cells with self-renewing capabilities that may play important roles in the establishment of T cell memory subsets [76]; observations such as these are likely to also play key roles to guide the development of the next generation of biomarkers to evaluate in T cell therapy trials.

Multi-parametric analyses that combine the evaluation of surface and activation markers with effector function markers such as CD107a/b, perforin and granzyme, intracellular detection of effector cytokines such as IL-2, IFN-γ, TNF-α, IL-4, MIP-a, MIP1B, and concomitantly the phosphorylation status of intracellular signaling molecules important for T cell function [77,78] afford the potential, still largely untapped, to evaluate directly ex-vivo T cell functional competence and identify treatment and outcome relevant biomarkers.

As discussed above, recently described novel high-throughput and deep sequencing technologies afford the opportunity to evaluate in a systematic and essentially comprehensive manner the T cell repertoire diversity directly ex-vivo [56,57]. Such approaches, combined with tools such as those described above to enrich for defined T cell subsets and specificities, have the potential to revolutionize the ability for insights into the biomarker signature(s) associated with clinically relevant T cell bioactivity.

Finally, important insights about the relevant biomarkers to evaluate with regard to T cell phenotypes and function can be derived from the characterization and release testing associated with product manufacture. In particular, well defined and robust assays for product identity and potency that measure relevant functional parameters for the products can provide valuable information about the properties of the cell product, as well as help establish and qualify the assays that will be used on the clinical samples.

#### iii. Biomarkers to evaluate T cell bioactivity

Insights about product bioactivity can often be obtained by evaluating the impact of the treatment on patient biology. A classic example of this is the delayed-type hypersensitivity (DTH) reaction observed at the site of injection, which is associated with an injection-mediated inflammatory reaction. Autoimmune vitiligo associated with the destruction of normal melanocytes has been reported to be associated with anti-tumor activity following melanoma T cell immunotherapy [79]. More recently significant off -tumor-target antigen-specific autoimmunity was observed when T cells specific for antigens expressed by normal tissues were transferred to patients [80-82]. These unfortunate results have revealed at least some of the pitfalls associated with the potency of T cell therapy-based clinical strategies, and underscore the urgent need to identify and develop early biomarker signatures to track these non-desired consequences of T cell therapy-based strategies. Cytokine analyses of serum samples obtained pre- and post- treatment appear to be particularly useful in this regard: such analyses have revealed evidence for a pre-infusion elevated cytokine milieu (elevation of IL-2, IL-7, IL-15, and IL-12) in one case [82], and evidence for severe cytokine storm post infusion T cell infusion in another case; cytokine storm was associated with elevated levels of the factors IFN-γ, GM-CSF, TNF-α, IL-6, and IL-10 [81]. These observations have prompted a movement for real-time assessment of systemic levels for the above cytokines in patients during treatment, particularly when cytokine-storm related symptoms are observed. Such real-time cytokine assessment was recently applied and used to support the documentation of delayed (22 days post T cell infusion) tumor lysis syndrome in a CLL patient with advanced treatment-refractory disease following infusion of T cells modified to express a CAR that targeted CD19. The delayed tumor lysis syndrome in this patient was diagnosed on the basis of significant elevations in uric acid, phosphorus, and lactate dehydrogenase as well as evidence of acute kidney injury with elevated creatinine levels, and was paralleled by robust in vivo expansion of CAR-modified cells and dramatic but transient increases in systemic levels for a number of pro-inflammatory cytokines and chemokines and a rapid and robust clinical response [41]. A related recent report describes the use of multiplex bead array technology to monitor in a systematic manner the modulation of a collection of 30 cytokines, chemokines, and growth factors in peripheral blood and marrow samples from CLL patients treated with CD19 CAR modified T cells; these studies showed transient modulation for a number of factors that coincided with peak T cell proliferation and activity, followed by return to baseline values despite long-term persistence and functionality of infused modified cells [40].

The development of new systems biology-based platforms has provided the opportunity to query the impact of T cell bioactivity on patient biology at a broader level. Such platforms, which have not yet been extensively applied to T cell therapy trials, include molecular array-[83,84] and proteomics- [85,86] based analyses, as well as high throughput multiplex-bead array based assays to measure changes in cytokine, chemokine, and other immune factors in patients post-T cell infusion. The systematic application of these and other systems-biology-based platforms has the potential to provide fundamental and unprecedented insights into molecular, secreted and functional biomarkers that correlate with T cell bioactivity and effective anti-tumor immunity.

#### iv. Biomarkers to evaluate patient immune responses to the infused T cells

In essentially all to-date clinical trials, T cell products are manipulated ex-vivo prior to infusion into patients. The primary objective of such manipulations is to enhance the potency of the product by increasing T cell numbers through culture and/or to endow T cells with novel/enhanced anti-tumor functionalities. In the context of autologous T cell transfer, many of these manipulations also have the potential to make the T cell immunogenic following transfer. The move away from xenobiotic sera and toward using serum-free formulations for T cell expansion cultures has minimized a major source of potential immunogenicity attributable to the manufacturing process. Two major potential sources of immunogenicity are related to the genetic engineering required to endow T cells with enhanced anti-tumor functionality. The first source of potential immunogenicity is the existence of non-self translated open reading frames expressed by the vector. Such open reading frames can be intentional, for example to express non- human gene products such as neomycin phosphotransferase which allow selection for gene-modified cells and the HyTK fusion protein which allows for both selection of modified cells and, by virtue of the thymidine kinase (TK) gene product, in-vivo selection against infused cells. Anti-transgene cellular immune responses to such selectable gene products which mediate T cell rejection have been demonstrated in a number of cases using both in-vitro culture and expansion [87] as well as directly ex vivo using a combination of Vβ spectratyping and CD107 degranulation [55]. The second source of potential immunogenicity is a result of the use of murine antibody scFv determinants and the creation of unique junctional fragments in the design of chimeric antigen receptors; recent reports describes the generation of both humoral and in one case cellular immune responses that target CAR sequence determinants as well the generation of cellular immune responses against what were presumably epitopes derived from the retrovirus vector backbone; detection of these responses was associated with disappearance of infused cells from the peripheral circulation [88,89]. Since the generation of anti-infused T cell immunity has profound implications for T cell persistence, such analyses ought to be considered an essential component of T cell biomarker studies.

## Conclusions

The significant potential of T cell immunotherapy as an effective approach to target cancer is beginning to be realized in a number of clinical settings. As discussed above, a wide variety of biomarkers have been developed and are available to evaluate T cell bioactivity. Since it is unlikely that clinical efficacy of T cell immunotherapy based approaches will be causally associated with a single biomarker, a major challenge for the field will be to establish the infrastructure to support biomarker analyses that are as comprehensive and broad as possible, and driven by principles of quality [9]. Development of this infrastructure needs to specifically be supported by the following elements:

A. The development and integration into T cell biomarker studies of assay platforms that are more sensitive and capable of higher complexity analyses. In this regard, array and other high throughput analysis based platforms that can evaluate large panels of nucleic acid or protein biomarkers are likely to be particularly useful.

B. The establishment of quality infrastructure and operations in laboratories that perform T cell biomarker analyses to facilitate the generation and collection of robust data sets that can be applied to generate statistically meaningful conclusions from relatively small cohorts and samples sets.

C. The development of algorithms and programs that allow for the multi-factorial and/or Boolean analyses of the data, as described elegantly by a number of groups [59,60,90,91], that will enable a more systems biology-based analysis of biomarker data sets generated in T cell therapy trials.

D. As recommended by the minimum reporting guidelines consortium(MIBBI) [92], The development and implementation of appropriate annotation and storage of data in repositories that can be openly accessed by the research community to facilitate more detailed and cross-study prospective or retrospective analyses of data. In particular for T cell therapy-based trials, the MIATA (Minimum Information About T-cell Assays) initiative has been established to specifically facilitate the identification of the relevant parameters important to document and report about T cell assays [93].

Establishment and implementation of the above elements may ultimately allow for the identification of product biomarker combinations that causally correlate with efficacy and therefore can be developed as surrogate endpoints of both outcome-and efficacy-relevant product bioactivity.

## List of abbreviations

None

## Competing interests

The author declares that they have no competing interests.
